# Further Insights on the Carotenoid Profile of the Echinoderm *Marthasterias glacialis* L

**DOI:** 10.3390/md10071498

**Published:** 2012-07-12

**Authors:** Lilian R. B. Mariutti, David M. Pereira, Adriana Zerlotti Mercadante, Patrícia Valentão, Natércia Teixeira, Paula B. Andrade

**Affiliations:** 1 Department of Food Science, Faculty of Food Engineering, University of Campinas (UNICAMP), Rua Monteiro Lobato, 80, CEP 13083-862, Campinas, São Paulo, Brazil; Email: lilianmariutti@gmail.com; 2 REQUIMTE/Laboratory of Pharmacognosy, Department of Chemistry, Faculty of Pharmacy, University of Porto, R. de Jorge Viterbo Ferreira, 228, 4050-313 Porto, Portugal; Email: david.ffup@gmail.com (D.M.P.); valentao@ff.up.pt (P.V.); 3 Laboratory of Biochemistry, Department of Biological Sciences, Faculty of Pharmacy, University of Porto, R. de Jorge Viterbo Ferreira, 228, 4050-313 Porto, Portugal; Email: natercia@ff.up.pt; 4 IBMC—Institute for Cell and Molecular Biology, University of Porto, 4150-180 Porto, Portugal

**Keywords:** *Marthasterias glacialis* L., carotenoids, isomers, echinoderm, HPLC-DAD-APCI-MS/MS

## Abstract

In this study, the carotenoid profile of the echinoderm *Marthasterias glacialis* L. was established using HPLC-DAD-APCI-MS/MS equipped with a C_30_ column. This approach rendered the identification of 20 compounds, eight of them reported for the first time in this marine organism. Differentiation of carotenoid isomers was also achieved.

## 1. Introduction

Carotenoids constitute a class of isoprenoids widespread in nature, with over 700 compounds documented to this day [1]. These metabolites are responsible for the colors of some plants, algae, microbes and also some animals. For many years, this class of molecules was thought to be present in animals solely as a result of predation upon organisms from lower trophic levels. However, in 2010 the first carotenoid-synthesizing animal, the pea aphid, was described and is thought to be a result of lateral gene transfer in a context of co-evolution [2].

In plants, carotenoids act as antioxidants, regulators of membrane fluidity and as light-harvesting molecules in photosynthetic systems [3]. In terms of human health, these metabolites are very important due to their strong antioxidant [4] and provitamin A activity displayed by some compounds of this group [5]. A recent meta-analysis showed that a diet rich in carotenoids was effective in the prevention of late age-related macular degeneration [6], although the contribution of these molecules to the treatment of this pathology requires further study.

The presence of carotenoids in the echinoderm *Marthasterias glacialis* L. (spiny sea star) was reported in the 1970’s [7]. At the time, several carotenoids had been identified, such as lutein, zeaxanthin and astaxanthin. While the presence of these compounds was recently confirmed, other compounds that have been previously reported, such as echinenone and 5,6-epoxy lutein, were not found [8]. In both works, no characterization of carotenoid isomers was attempted.

Atmospheric pressure chemical ionization (APCI) forms abundant positively or negatively charged molecular ions or protonated and deprotonated molecules of both carotenes and xanthophylls, and their fragmentation pattern can also help with the identification of this type of compounds. In the present work, we deepen the study of the carotenoid composition of *M. glacialis* using a C_30_ column in a high performance liquid chromatography-diode array detector-mass spectrometry detector (HPLC-DAD-APCI-MS/MS) system. Using this type of column, it is possible to separate some of the carotenoid isomers that cannot be resolved with most C_18_ columns. Furthermore, given the fact that some carotenoid isomers are not commercially available, we have conducted isomerization and reduction procedures starting from available standards, in order to be able to confirm the identity of some peaks.

## 2. Results and Discussion

**Figure 1:**
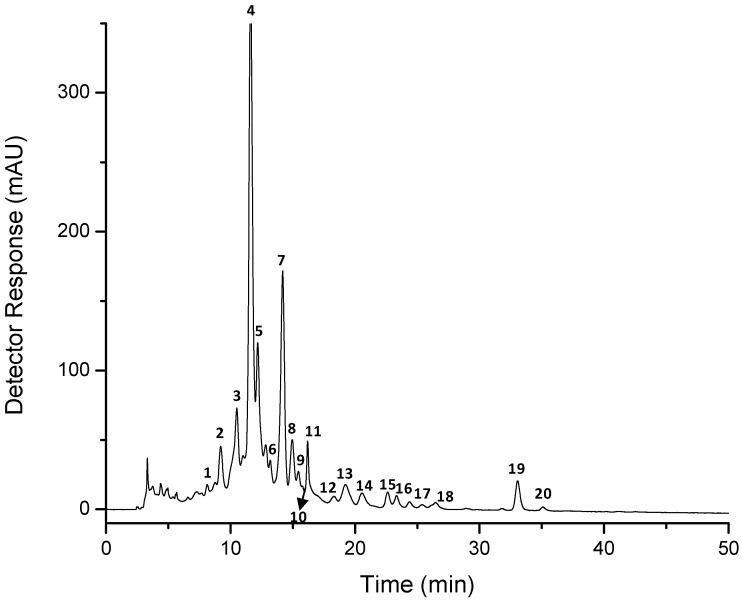
Chromatogram (processed at 450 nm), obtained by HPLC-DAD, of the carotenoids from *Marthasterias glacialis*. See text for chromatographic conditions. Peak identification and characterization are given in Table 1.

The carotenoids, extracted from the echinoderm *M. glacialis*, were chromatographically separated and subsequently tentatively identified, based on the combined information obtained from chromatographic elution, UV-vis and mass spectra characteristics (Table 1). The HPLC chromatogram (Figure 1) demonstrates the presence of 20 carotenoids in the extract of the spiny sea star. Since a detailed description of carotenoid identification using the above information is available in literature [9], only considerations regarding carotenoids not identified in previous reports, along with the mass spectroscopy data obtained by negative APCI, are discussed in the present work.

**Table 1:** Chromatographic, UV-vis and mass spectrometry characteristics of carotenoids from *Marthasterias glacialis* (spiny sea star), obtained by HPLC-DAD-MS/MS.

Peak ^a^	Carotenoid	*t*_R_^ b^(min)	λ_max_^ c^ (nm)	% III/II	[M + H]^+^ (*m/z*)	MS/MS fragment ions (positive mode) (*m/z*)	M^−•^ or [M − H]^−^ (*m/z*)	MS/MS fragment ions (negative mode) (*m/z*)
**1**	not identified 1	8.7–8.8	419, 445, 469	nc ^d^	597	579 [M + H − 18]^+^, 561 [M + H − 18 − 18]^+^, 505 [M + H − 92]^+^	595 ^f^	577 [M − H − 18]^−^, 559 [M − H − 18 − 18]^−^
**2**	crustaxanthin	9.1–9.2	422, 449, 470	nc	601	583 [M + H − 18]^+^	599 ^f^	581 [M − H − 18]^−^, 563 [M − H − 18 − 18]^−^, 507 [M − H − 92]^−^
**3**	13-*cis*-astaxanthin	10.4–10.5	371, 466	0	597	579 [M + H − 18]^+^, 561 [M + H − 18 − 18]^+^, 379, 285, 173	596 ^g^	581 [M − 15]^−^, 578 [M − 18]^−^, 504 [M − 92]^−^, 429 [M − 167]^−^, 389, 363 [M − 233]^−^, 337 [M − 167 − 92]^−^, 323, 297, 233
**4**	all-*trans*-astaxanthin	11.2–11.6	475	0	597	579 [M + H − 18]^+^, 561 [M + H − 18 − 18]^+^, 505 [M + H − 92]^+^, 379, 285	596 ^g^595 ^f^	581 [M − 15]^−^, 577 [M − H − 18]^−^, 559 [M − H − 18 − 18]^−^, 541 [M − H − 18 − 18 − 18]^−^, 504 [M − 92]^−^, 429 [M − 167]^−^, 490 [M − 106]^−^, 389, 363 [M − 233]^−^, 337 [M − 167 − 92]^−^, 323, 297, 233, 203
**5**	all-*trans*-lutein	11.9–12.2	421, 445, 472	67–71	nd ^e^	551 ^h^ [M + H − 18]^+^, 533 [M + H − 18 − 18]^+^, 495 [M + H − 18 − 56]^+^, 459 [M + H − 18 − 92]^+^, 430, 175	568 ^g^567 ^f^	549 [M − H − 18]^−^, 531 [M − H − 18 − 18]^−^, 535 [M − 18 − 15]^−^, 429
**6**	astaxanthin derivative 1	13.0–13.2	472	0	597	579 [M + H − 18]^+^, 561 [M + H − 18 − 18]^+^, 379, 285, 173	596 ^g^	581 [M − 15]^−^, 578 [M − 18]^−^, 542 [M − 18 − 18 − 18]^−^, 429 [M − 167]^−^, 490 [M − 106]^−^, 389, 363 [M − 233]^−^, 337 [M − 167 − 92]^−^, 323, 297, 233, 203
**7**	all-*trans*-zeaxanthin	13.8–14.2	423, 451, 476	25–40	569	551 [M + H − 18]^+^, 533[M + H − 18 − 18]^+^, 459, 416, 175	567 ^f^	549 [M − H − 18]^−^, 534 [M − H − 18 − 15]^−^, 531 [M − H − 18 − 18]^−^, 465, 201, 187
**8**	astaxanthin derivative 2	14.8–15.0	459	nc	597	579 [M + H − 18]^+^, 561 [M + H − 18 − 18]^+^, 285, 173	595 ^f^596 ^g^	581 [M − 15]^−^, 577 [M−H − 18]^−^, 429 [M − 167]^−^, 389, 363 [M − 233]^−^, 337 [M − 167 − 92]^−^, 323, 297, 233, 203
**9**	9-*cis*-astaxanthin	15.5	470	0	597	579 [M + H − 18]^+^, 561 [M + H − 18 − 18]^+^	596 ^g^	581 [M − 15]^−^, 578 [M − 18]^−^, 560 [M − 18 − 18]^−^, 504 [M − 92]^−^, 429 [M − 167]^−^, 490 [M − 106]^−^, 363 [M − 233]^−^, 337 [M − 167 − 92]^−^, 323, 297, 233, 203
**10**	astaxanthin derivative 3	16.2–16.4	454, 475	nc	597	579 [M + H − 18]^+^, 379, 285	596 ^g^	581 [M − 15]^−^, 578 [M − 18]^−^, 504 [M − 92]^−^, 429 [M − 167]^−^, 490 [M − 106]^−^, 389, 337 [M − 167 − 92]^−^, 233
**11**	not identified 2	16.8	422, 452, 472	nc	565	nd	564 ^g^	nd
**12**	all-*trans*-canthaxanthin	18.3	470	0	565	nd	564 ^g^	nd
**13**	5,6-epoxy-β-cryptoxanthin	19.2	419, 447, 472	67	569	551 [M + H − 18]^+^, 221	567 ^f^	549 [M − H − 18]^−^
**14**	not identified 3	20.5–20.7	423, 452, 478	20–25	601	585, 548, 507, 441, 413	600 ^g^	581, 543, 416
**15**	all-*trans*-β-cryptoxanthin	22.6	422, 450, 476	25	nd	nd	552 ^g^	534 [M − 18]^−^, 519 [M − 18 − 15]^−^, 269, 243
**16**	not identified 4	23.3	422, 450, 472	25	nd	nd	nd	nd
**17**	15-*cis*-β-carotene	25.4	420, 448, 471	nc	537	413 [M + H − 124]^+^	536 ^g^	295, 269, 189
**18**	13-*cis*-β-carotene	26.5	418, 447, 471	nc	537	269	536 ^g^	444 [M − 92]^−^, 295, 269
**19**	all-*trans*-β-carotene	33.1–34.7	422, 451, 477	20	537	444 [M + H − 92]^+^, 413 [M + H − 124]^+^, 400 [M + H − 137]^+^, 269, 177	536 ^g^	444 [M − 92]^−^, 295, 269
**20**	9-*cis*-β-carotene	35.1–36.8	419, 448, 472	nc	537	444 [M + H − 92]^+^, 269	535 ^f^	295, 269

^a^ Numbered according to Figure 1; ^b^ Retention time on the C_30_ column; ^c^ Linear gradient methanol/MTBE; ^d^ Not calculated; ^e^ Not detected; ^f^ [M − H]^−^; ^g^ M^−•^; ^h^ Fragmentation in source.

As carotenoids are well-known to form stable protonated molecules ([M + H]^+^) upon positive ionization, the sample was firstly analyzed in the positive ion mode. As expected, for most of the peaks, the protonated molecule, as well as the respective fragment ions formed, both from the polyene chain and functional groups, were generated. However, an extremely high background noise, probably originating from residual lipids present in the sample also forming positive ions, impaired the identification of the protonated molecules or the interpretation of the fragmentation pattern even by MS/MS. As an example, a very intense signal at *m/z* 369, which corresponds to cholesterol [M + H]^+^, could be observed from 16 to 20 min. Therefore, the sample was also analyzed in the negative mode and under this condition, negatively charged molecular ions (M^−•^) were formed, whilst deprotonated molecules [M − H]^−^ were formed in just a few cases. The formation of M^−•^ in the negative mode has previously been described for lutein, α-carotene, β-carotene [10] and astaxanthin esters [11].

**Figure 2:**
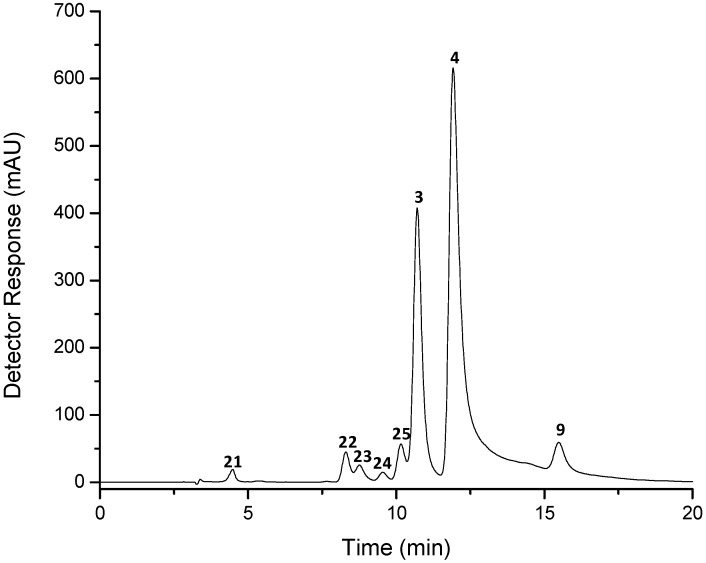
Chromatogram (processed at 450 nm), obtained by HPLC-DAD, of astaxanthin standard submitted to heat under reflux. See text for chromatographic conditions. Peak identification and characterization are given in Table 2.

The thermal-induced isomerization of all-*trans*-astaxanthin standard solution was carried out in order to indicate the presence of *cis* isomers in the sample. The chromatographic separation of the heated astaxanthin standard solution on the C_30_ column is shown in Figure 2. The relative amounts of the eight compounds derived from all-*trans*-astaxanthin were 1.0% of apo-10′-astaxanthinal, 2.8%, 1.7% and 0.6% of different di-*cis*-isomers of astaxanthin, 3.2% of mono-*cis*-astaxanthin, 29.2% of 13-*cis*-astaxanthin, 50.2% of all-*trans*-astaxanthin and 3.9% of 9-*cis*-astaxanthin. The peaks were tentatively identified based on spectral features, chromatographic elution, mass spectra characteristics (Table 2) and literature data [12,13,14,15]. Moreover, four oxidation products of astaxanthin were detected by the extracted ion chromatograms obtained at *m/z* 315, *m/z* 341, *m/z* 381 and *m/z* 447, corresponding to apo-15′-astaxanthinal (13.3 min), apo-14′-astaxanthinal (6.7 min), apo-12′-astaxanthinal (4.2 min) and apo-8′-astaxanthinal (5.6 min), respectively. When the *trans-*form of carotenoids is isomerized to the *cis*-isomers, a small and large hypsochromic shift in the absorption maximum for, respectively, mono-*cis* carotenoids and di-*cis* carotenoids occurs, as well as the intensity of the *cis*-peak, which increases as the *cis* double bond approaches the centre of the molecule [16]. Peak 4 corresponds to all-*trans*-astaxanthin and the two main isomers formed were assigned as 13-*cis*-astaxanthin (peak 3) and 9-*cis*-astaxanthin (peak 9), with hypsochromic shifts of 8 and 5 nm, respectively, whilst an additional absorption band was present at 370 nm for the 13-*cis* isomer. These characteristics are in agreement with the data presented by Holtin *et al.* [14], which confirmed the identity of these two isomers by nuclear magnetic resonance (NMR). Peak 25 was identified as a *cis-*astaxanthin and presents similar UV-vis characteristics to 13-*cis*-astaxanthin, *i.e.*, hypsochromic shift of 7 nm and a *cis*-peak at 368 nm, but it is probably not a 15-*cis* isomer since it should present a more intense *cis*-peak than 13-*cis* isomer. Apart from peak 21 (*m/z* 406) and 23 (no molecular ion was detected), all the other compounds presented in Figure 2 (peaks 3, 4, 9, 22, 24 and 25) showed the molecular ion (M^−•^) at *m/z* 596 when analyzed by negative ion APCI. However, by positive ion mode, the protonated molecule at *m/z* 597 was present only in the mass spectra of peaks 3, 4, 9 and 25. Peaks 22 to 24 presented hypsochromic shifts between 17 and 21 nm, relative to the absorption maxima of the all-*trans* isomer, and were identified as di-*cis*-astaxanthin, based on comparison with literature data [14]. Peak 21 was identified as apo-10′-astaxanthinal, which is an oxidation product of astaxanthin with a molecular weight of 406 Da. The MS spectra of apo-10′-astaxanthinal presented the protonated molecule at *m/z* 407 [M + H]^+^ in positive ion mode and the molecular ion at *m/z* 406 (M^−•^) in negative ion mode. In addition, in both ionization modes, the MS/MS fragments corresponded to the loss of one hydroxyl group (18 u). 

**Table 2:** Chromatographic, UV-vis and mass spectrometry characteristics of astaxanthin standard submitted to heat under reflux, obtained by HPLC-DAD-MS/MS.

Peak ^a^	Carotenoid	*t*_R_^b^ (min)	λ_max_^ c^ (nm)	Δλ	% III/II	% A_B_/A_II_	[M + H]^+^ (*m/z*)	MS/MS fragment ions (positive mode) (*m/z*)	M^−•^ (*m/z*)	MS/MS fragment ions (negative mode)(*m/z*)
**21**	apo-10′-astaxanthinal	4.4–4.5	428	48	0		407	389 [M + H − 18]^+^	406	388 [M − 18]^−^
**22**	di-*cis*-astaxanthin 1	8.2–8.3	457	19	0		nd ^d^	nd	596	578 [M − 18]^−^, 564 [M − 18 − 18]^−^, 504 [M − 92]^−^
**23**	di-*cis*-astaxanthin 2	8.6–8.8	455–457	21	0		nd	nd	nd	nd
**24**	di-*cis*-astaxanthin 3	9.5	459–457	17	0		nd	nd	596	nd
**25**	*cis*-astaxanthin	10.2–10.3	368, 469	7	0	58	597	285	596	581 [M − 15]^−^, 578 [M − 18]^−^, 504 [M − 92]^−^, 429 [M − 167]^−^, 389, 363 [M − 233]^−^, 337 [M − 167 − 92]^−^, 233
**3**	13-*cis*-astaxanthin	10.7–10.8	370, 468	8	0	51	597	579 [M + H − 18]^+^, 561 [M + H − 18 − 18]^+^, 379, 285, 173	596	581 [M − 15]^−^, 578 [M − 18]^−^, 504 [M − 92]^−^, 429 [M − 167]^−^, 389, 363 [M − 233]^−^, 337 [M − 167 − 92]^−^, 233
**4**	all-*trans*-astaxanthin	11.8–11.9	476		0		597	579 [M + H − 18]^+^, 505 [M + H − 92]^+^, 379, 285, 173	596	581 [M − 15]^−^, 578 [M − 18]^−^, 504 [M − 92]^−^, 429 [M − 167]^−^, 389, 363 [M − 233]^−^, 337 [M − 167 − 92]^−^, 297, 233, 167
**9**	9-*cis*-astaxanthin	15.5–15.7	471	5	0		597	579 [M + H − 18]^+^, 561 [M + H − 18 − 18]^+^, 379, 285	596	581 [M − 15]^−^, 578 [M − 18]^−^, 504 [M − 92]^−^, 429 [M − 167]^−^, 389, 363 [M − 233]^−^, 337 [M − 167 − 92]^−^, 233

^a^ Numbered according to Figure 2; ^b^ Retention time on the C_30_ column; ^c^ Linear gradient methanol/MTBE; ^d^ Not detected.

**Table 3:** Chromatographic, UV-vis and mass spectrometry characteristics of astaxanthin standard submitted to reduction with NaBH_4_, obtained by HPLC-DAD-MS/MS.

Peak ^a^	Carotenoid	*t*_R _^b^ (min)	λ_max_^ c^ (nm)	% III/II	% A_B_/A_II_	[M + H]^+ ^(*m/z*)	MS/MS fragment ions (positive mode) (*m/z*)	[M − H]^−^ (*m/z*)	MS/MS fragment ions (negative mode)(*m/z*)
**26**	crustaxanthin	7.9	422, 449, 476	40	0	601	583 [M + H − 18]^+^, 565 ^d^ [M + H − 18 − 18]^+^, 547 [M + H − 18-18-18]^+^, 509 [M + H − 92]^+^	599	581 [M − 18]^−^, 563 [M − 18 − 18]^−^, 545 [M − 18 − 18 − 18]^−^, 507 [M − 92]^−^, 493 [M − 106]^−^
**27**	crustaxanthin	8.5	422, 449, 476	35	0	601	583 [M + H − 18]^+^, 565 [M + H − 18 − 18]^+^, 547 ^d^ [M + H − 18 − 18 − 18]^+^, 509 [M + H − 92]^+^	599	581 [M − 18]^−^, 563 [M − 18 − 18]^−^, 545 [M − 18 − 18 − 18]^−^, 507 [M − 92]^−^
**28**	*cis*-crustaxanthin	9.0	337, 422, 449, 476	40	6	601	583 [M + H − 18]^+^, 565 [M + H − 18 − 18]^+^, 547 [M + H − 18 − 18 − 18]^+^, 509 [M + H − 92]^+^	599	581 [M − 18]^−^, 563 [M − 18 − 18]^−^, 545 [M − 18 − 18 − 18]^−^, 507 [M − 92]^−^
**29**	*cis*-crustaxanthin	9.3	337, 420, 444, 470	20	39	601	583 [M + H − 18]^+^, 565 [M + H − 18 − 18]^+^, 547 [M + H − 18 − 18 − 18]^+^, 509 [M + H − 92]^+^	599	581 [M − 18]^−^

^a^ Numbered according to Figure 3; ^b^ Retention time on the C_30_ column; ^c^ Linear gradient methanol/MTBE; ^d^ Fragmentation in source.

**Figure 3:**
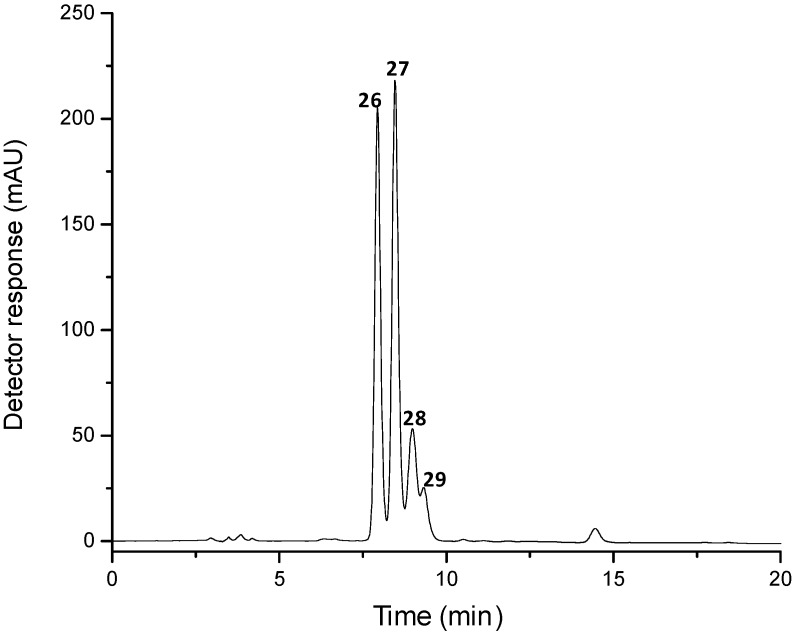
Chromatogram (processed at 450 nm), obtained by HPLC-DAD, of astaxanthin standard submitted to reduction with NaBH_4_. See text for chromatographic conditions. Peak identification and characterization are given in Table 3.

Crustaxanthin was synthesized by astaxanthin reduction with NaBH_4_, and the result of this reaction was separated on C_30_ column, as shown in Figure 3. Four crustaxanthin isomers were separated; however, by chromatographic and MS data we could only tentatively differentiate among *trans* and *cis* isomers, since this carotenoid has four asymmetric carbons and thus 10 different optical *R*/*S* isomers. The peaks were tentatively identified, based on spectral features, chromatographic elution, mass spectra characteristics (Table 3) and literature data [1]. The four peaks presented protonated molecule at *m/z* 601 and deprotonated molecule at *m/z* 599, which indicates that both keto groups of astaxanthin were reduced to hydroxyl groups. The fragmentation pattern was also similar for all the crustaxanthin isomers with fragments corresponding to the loss of one (18 u), two (36 u) and three (54 u) hydroxyl groups, as well as toluene (92 u) and xylene (106 u) from the polyene chain, both in the positive and negative fragmentation ion modes. Peaks 28 and 29 were assigned as *cis*-crustaxanthin due to the presence of a *cis*-peak at 337 nm.

In the spiny sea star (Figure 1, Table 1), the astaxanthin isomers*,* all-*trans*-astaxanthin (peak 4), 13-*cis*-astaxanthin (peak 3) and 9-*cis*-astaxanthin (peak 9) were identified considering the UV-vis spectra characteristics, chromatographic behavior, co-elution with isomerized standard (Table 2), and mass spectra. The mass spectra of all isomers of astaxanthin obtained in the positive ion mode showed the protonated molecule at *m/z* 597 and fragment ions in the MS/MS at *m/z* 579 [M + H − 18]^+ ^and *m/z* 561 [M + H − 18 − 18]^+^, corresponding to the loss of one and two hydroxyl groups, respectively. However, the fragment at *m/z* 505 [M + H − 92]^+^, resulting from the loss of toluene from the polyene chain, was detected only in the all-*trans*-astaxanthin spectra of both sample and standard. In the low mass region of the positive ion APCI tandem mass spectra of astaxanthin isomers, fragment ions corresponding to cleavages of the polyene chain were detected at *m/z* 173, *m/z* 285 and *m/z* 379 [15]. In the negative ion mode, the molecular ion at *m/z* 596 was present in the MS spectra of all the isomers and only all-*trans*-astaxanthin presented the deprotonated molecule at *m/z* 595 with the same intensity as the molecular ion. In addition, the MS/MS showed the presence of fragments at *m/z* 581 [M − 15]^−^, *m/z* 578 [M − 18]^−^, *m/z* 560 [M − 18 − 18]^−^, *m/z* 504 [M − 92]^− ^or *m/z* 490 [M − 106]^−^, resulting from the losses of a methyl radical, one and two hydroxyl groups, toluene and xylene, respectively, and also the presence of other fragments from the polyene chain cleavage previously reported by van Breemen *et al.* [15] at *m/z* 203, *m/z* 233, *m/z* 297, *m/z* 323, *m/z* 337 [M − 167 − 92]^−^, *m/z* 363 [M − 233]^−^, *m/z* 389 and *m/z* 429 [M − 167]^−^. The fragment ion at *m/z* 233 and its complementary ion at *m/z* 363 [M − 233]^−^ were detected in all the MS/MS spectra of the astaxanthin isomers, and correspond to the cleavage of the 11,12 carbon-carbon bond with hydrogen transfer from the leaving group to the ion. The fragment at *m/z* 167, corresponding to the cleavage of the 7,8-bond with hydrogen transfer from the leaving group to the ion, was not detected; however, its complementary ion at *m/z* 429 [M − 167]^−^ and the ion at *m/z* 337 [M − 167 − 92]^−^, which corresponds to the loss of toluene from the ion of *m/z* 429, were detected in all the MS/MS spectra of the astaxanthin isomers.

Peak 2 (Figure 1, Table 1) was identified as crustaxanthin (3,4,3′,4′-tetrahydroxy-β,β-carotene), an astaxanthin metabolite, since it presented the same mass spectra of characteristics peaks 28 and 29 and UV-vis characteristics of peak 29 of the reduced astaxanthin chromatogram (Figure 3, Table 3). All-*trans*-canthaxanthin (peak 12) (Figure 1, Table 1), a carotenoid commonly used as a fish and poultry feed supplement, was identified, based on its UV-vis characteristics, molecular weight (564 Da) obtained from mass spectroscopic data, elution order on the C_30_ column and co-elution with an authentic standard. Peaks 6, 8 and 10 (Figure 1, Table 1) possess the same molecular weight and lack the fine structure as does astaxanthin (596 Da), thus they were assigned as astaxanthin derivative 1, 2 and 3, respectively. The lack of a fine structure is an indicator for the conjugation of a carbonyl group with the chromophore. 

All-*trans*-lutein (peak 5) and all-*trans*-zeaxanthin (peak 7) (Figure 1, Table 1) have the same chemical formula (C_40_H_56_O_2_) and, consequently, identical protonated (*m/z* 569), deprotonated (*m/z* 567) molecules and molecular ions (*m/z* 568). The structural difference between these carotenoids is that zeaxanthin has two β-ring end-groups, while lutein has one β-ring and one ε-ring. Therefore, zeaxanthin has both double bonds in the β-ring conjugated to the polyene chain, resulting in a chromophore with eleven conjugated double bonds, and lutein has one hydroxyl allylic to the double bound in the ε-ring that is not conjugated to the polyene chain, resulting in a chromophore with 10 conjugated double bonds. These characteristics allow differentiation between these two carotenoids, based on both UV-vis and mass spectra. As expected, zeaxanthin showed higher λ_max_ values than lutein. In addition, zeaxanthin mass spectrum in positive ion mode presented higher intensity of the protonated molecule peak (569 u) in comparison to the fragment at *m/z* 551 [M + H − 18]^+^, which indicates that the hydroxyl group is not allylic to the double bond, whilst in the lutein spectrum the fragment at *m/z* 551 was more intense than the protonated molecule at *m/z* 569, as previously reported in the literature [1,9,17]. The MS/MS spectra obtained in the positive ion mode showed fragments at *m/z* 533 [M + H − 18 − 18]^+^, *m/z* 495 [M + H − 18 − 56]^+^, *m/z* 459 [M + H − 18 − 92]^+^, similarly to data reported in the literature [1,9,17]. In the negative ion mode, the MS/MS spectrum of lutein presented a fragment at *m/z* 429, which corresponds to the elimination of the terminal ring containing the unconjugated carbon-carbon double bond, and consequently can be used to distinguish between lutein and zeaxanthin [15]. The identity of both lutein and zeaxanthin was confirmed by co-elution with authentic standards. 

All-*trans*-β-cryptoxanthin (peak 15) (Figure 1, Table 1) presented a similar UV-vis spectrum than that of all-*trans*-β-carotene and that of all-*trans*-zeaxanthin, since they possess the same chromophore. The identification of this carotenoid was also based on its molecular ion at *m/z* 552 obtained in the negative ion mode and the MS/MS fragments at *m/z* 534 [M − 18]^−^, *m/z* 519 [M − 18 − 15]^−^, *m/z* 269 and *m/z* 243, since the protonated molecule was not detected in the positive ion mode. The identification was confirmed by co-elution with authentic standard. Peak 13 was assigned as 5,6-epoxy-β-cryptoxanthin, considering the UV-vis, the MS characteristics and comparison with literature data [1,9,18]. The presence of a mass fragment at *m/z* 221 in the positive ion mode, which corresponds to the location of the epoxide group in the 3-hydroxy-β-ring, allowed the identification of this carotenoid as the 5,6-epoxide and not the 5′,6′-epoxide.

Peaks 17, 18, 19 and 20 (Figure 1, Table 1) were identified as 15-*cis*-, 13-*cis*-, all-*trans*- and 9-*cis*-β-carotene, respectively, based on the elution order on the C_30_ column, UV-vis and mass spectra characteristics and literature data [1,9,15,17]. The mass spectra of β-carotene isomers, which were obtained in the negative ion mode, showed the molecular ion at *m/z* 536, except for 9-*cis*-β-carotene, that presented the deprotonated molecule at *m/z* 535. The β-carotene isomers were present in very low concentrations, so the typical fragment ion in the MS/MS resulting from the loss of toluene ([M − 92]^−^) was observed only for 13-*cis*- and all-*trans*-β-carotene; other fragments in the low mass range described in the literature at *m/z* 269 and *m/z* 295 [15] were detected for all isomers. The identity of all-*trans*-β-carotene was confirmed by co-elution with authentic standard.

Although it was not possible to identify the peaks 1, 11, 14 and 16 (Figure 1), these unknown compounds presented UV-vis spectra with characteristic typical for carotenoids, as can be seen in Table 1. 

Astaxanthin monoesters or diesters were not detected, since in the chromatographic conditions used, they should, respectively, elute between 20 min and all-*trans-*β-carotene elution (33.1 to 34.7 min) and after all-*trans-*β-carotene to 50 min. The protonated molecules or main mass ions from carotenoid esters where searched in the range of *m/z* 100 and *m/z* 1500, but not detected. In addition, previous studies have conducted alkaline hydrolysis in the extract of this organism, with no esters being found [8]. Other carotenoids usually found in marine echinoderms and algae, such as 7,8-didehydroastaxanthin, 7,8,7′,8′-tetradehydroastaxanthin, fucoxanthin and echinenone, were also searched by observing the UV-vis spectra and the main ions of the mass spectra, but were not detected. Short chain products of astaxanthin, *i.e.*, apo-8′-, apo-10′-, apo-12′-, apo-14′- and apo-15-astaxanthinal, were not noticed in the sea star.

Recently, all-*trans*-astaxanthin, all-*trans*-lutein and all-*trans*-zeaxanthin were found in methanol and acetone extracts from *M. glacialis* and the purified methanol extract was able to inhibit cell proliferation against rat basophilic leukemia RBL-2H3 cancer cell line [8]. Both β-carotene and cryptoxanthin derivatives were searched, however they were not found. The fact that the work presented herein confirmed the presence of β-carotene and cryptoxanthin points to a possible inter-individual variation of this organism’s chemical composition.

Among the 20 carotenoids found in this study, this was the first time that the occurrence of crustaxanthin, all-*trans*-canthaxanthin, two astaxanthin and three β-carotene *cis*-isomers and 5,6-epoxy-β-cryptoxanthin was reported in *M. glacialis*.

## 3. Experimental Section

### 3.1. Reagents and Standards

HPLC grade solvents, methanol and methyl *tert*-butyl ether (MTBE) were obtained from Merck (Darmstadt, Germany) or Mallinckrodt Baker (Philipsburg, NJ, USA). Ethyl acetate, methanol, methylene chloride, hexane and chloroform, all analytical grade, were from Synth (Diadema, SP, Brazil). The solvents and samples were filtered through Millipore membranes (Bedford, MA, USA) of 0.22 and 0.45 μm, respectively.

The standards of all-*trans*-astaxanthin, all-*trans*-canthaxanthin and all-*trans*-β-carotene were acquired from Sigma-Aldrich (St. Louis, MO, USA). Standards of all-*trans*-lutein, all-*trans*-zeaxanthin and all-*trans*-β-cryptoxanthin were provided by DSM Nutritional Products (Basel, Switzerland). All standards showed at least 95% purity, determined by HPLC-DAD.

### 3.2. Sample

*M. glacialis* individuals were collected from the rocky coast at Cabo Carvoeiro, west Portugal, in March 2009, placed on ice and immediately transported to the laboratory in ice-boxes. The macro-invertebrates were then cleaned and washed with sea water and kept at −20 °C, prior to their lyophilization in a Labconco 4.5 Freezone apparatus (Kansas City, MO, USA). The dried material was powdered before extraction. The sample corresponded to a mixture of three individuals.

### 3.3. Carotenoid Extraction

In order to avoid carotenoid degradation during analysis, the manipulation of samples and extracts was carried out under dim light and at a controlled room temperature (22 ± 3 °C). The carotenoids were extracted from 2 g of freeze-dried samples using a mortar and a pestle with the aid of glass beads (Sigma-Aldrich, 150–212 µm) to break the cell walls. A total of nine consecutive extractions were performed: four with 10 mL of ethyl acetate, three with 10 mL of methanol and two with 10 mL of methylene chloride. The solvent was separated from the sample using a refrigerated centrifuge (Allegra 64R, Beckman Coulter, Palo Alto, CA, USA) at 10 °C and 1100 *g* for 10 min. To avoid the possible presence of glass beads in the final extract, each extract was filtered through a 0.45 μm membrane and the solvent was immediately evaporated at room temperature under nitrogen flux to avoid isomerization. The further extracts were added to the same tube. The dry extract was stored in darkness and under a nitrogen atmosphere (99.9% purity) at −80 °C until HPLC analysis. The extraction was carried out in triplicate. Immediately before HPLC analysis, the extracts were dissolved in 500 μL of methanol/MTBE (70:30, v/v) and sonicated for 3 min.

### 3.4. Preparation of Reference Compounds

About 1 mg of all-*trans* astaxanthin authentic standard was dissolved in 4 mL of chloroform and hexane was used to make up the volume to 25 mL. The work solution was prepared by diluting a 10 mL aliquot to 100 mL in a volumetric flask with 4 mL of chloroform with the volume being made up with hexane. The concentration of the work solution, 2.5 µM, was spectrophotometrically (Agilent, Palo Alto, CA, USA) determined using the absorption coefficient for astaxanthin (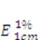 = 2100; 470 nm) [19]. 

To obtain the astaxanthin *cis* isomers, the work solution was mixed with 100 mL hexane and heated under reflux at boiling temperature (~69 °C) for 5 h. The solvent was evaporated in a rotary evaporator (Buchi, Flawil, Switzerland) and the isomerized dry standard was stored in darkness and under a nitrogen atmosphere (99.9% purity) at −36 °C until HPLC analysis. 

To synthesize crustaxanthin, the all-*trans* astaxanthin work solution was dried under nitrogen flux, redissolved in 10 mL of anhydrous ethanol and reduced with NaBH_4_ for 1h at room temperature [20]. The reaction was ended by adding saturated NaCl solution after which the carotenoids were extracted with dichloromethane. The solvent was removed under nitrogen flux and the dry crustaxanthin standard was stored in darkness and under a nitrogen atmosphere (99.9% purity) at −36 °C until HPLC analysis. 

### 3.5. HPLC-DAD-MS/MS Analysis

A Shimadzu HPLC (Kyoto, Japan) equipped with quaternary pumps (model LC-20AD), on-line degasser and a Rheodyne (Rheodyne LCC, Robert Park, USA) injection valve with a 20 µL loop, connected in series to a DAD detector (Shimadzu) and to a mass spectrometer with an ion trap analyzer and atmospheric pressure chemical ionization source (Bruker Daltonics, model Esquire 4000, Bremen, Germany) was used for all analysis. 

The carotenoids were separated on a C_30_ YMC column (3 μm, 250 × 4.6 mm i.d.) (Waters, Wilmington, USA) using as mobile phase a linear gradient of methanol/MTBE from 95:5 to 70:30 in 30 min, followed by 50:50 in 20 min. The flow rate was 0.9 mL·min^−1^ and column temperature was set at 29 °C. The UV-vis spectra were obtained between 250 and 650 nm and the chromatograms were processed at 450 nm. The MS parameters were set as follows: positive or negative mode; current corona: 4000 nA; source temperature: 450 °C; dry gas (nitrogen) temperature: 350 °C, flow: 5 L·min^−1^; nebulizer: 60 psi. The MS/MS was set in automatic mode, applying 1.4 V fragmentation energy. The mass spectra were acquired with an *m/z* range from 100 to 700 [9]. 

The identification of the carotenoids was performed, considering the combination of the following parameters: elution order on the C_30_ column, UV-vis spectrum features (maximum absorption wavelength (λ_max_), spectral fine structure (%III/II) and peak *cis* intensity (%A_B_/A_II_)), MS spectrum characteristics as compared to standards analyzed under the same conditions, co-chromatography with standards and data available in the literature. In addition, the assignment of the protonated molecule ([M + H]^+^) and the molecular ions (M^−•^) or deprotonated molecule ([M − H]^−^) was confirmed by second order MS fragmentation.

## 4. Conclusions

In this study, HPLC-DAD-APCI-MS/MS allowed the characterization of 20 carotenoids in the echinoderm *M. glacialis*, eight of them reported for the first time. Isomers of the main compounds identified before were reported. Overall, it seems that *M. glacialis* could be an interesting matrix for further studies as a source of antioxidant carotenoids with a positive impact in human health.
